# Evolutionary history of teleost intron-containing and intron-less rhodopsin genes

**DOI:** 10.1038/s41598-019-47028-4

**Published:** 2019-07-23

**Authors:** Chihiro Fujiyabu, Keita Sato, Ni Made Laksmi Utari, Hideyo Ohuchi, Yoshinori Shichida, Takahiro Yamashita

**Affiliations:** 1 Faculty of Science, Kyoto University, Kyoto, 606-8502 Japan; 2 Department of Cytology and Histology, Okayama University Graduate School of Medicine, Dentistry and Pharmaceutical Sciences, Okayama, 700-8558 Japan; 3 Department of Ophthalmology, Faculty of Medicine, Udayana University, Bali, Indonesia; 4 Research Organization for Science and Technology, Ritsumeikan University, Shiga, 525-8577 Japan; 5 Department of Biophysics, Graduate School of Science, Kyoto University, Kyoto, 606-8502 Japan

**Keywords:** Biochemistry, Evolution

## Abstract

Recent progress in whole genome sequencing has revealed that animals have various kinds of opsin genes for photoreception. Among them, most opsin genes have introns in their coding regions. However, it has been known for a long time that teleost retinas express intron-less rhodopsin genes, which are presumed to have been formed by retroduplication from an ancestral intron-containing rhodopsin gene. In addition, teleosts have an intron-containing rhodopsin gene (exo-rhodopsin) exclusively for pineal photoreception. In this study, to unravel the evolutionary origin of the two teleost rhodopsin genes, we analyzed the rhodopsin genes of non-teleost fishes in the Actinopterygii. The phylogenetic analysis of full-length sequences of bichir, sturgeon and gar rhodopsins revealed that retroduplication of the rhodopsin gene occurred after branching of the bichir lineage. In addition, analysis of the tissue distribution and the molecular properties of bichir, sturgeon and gar rhodopsins showed that the abundant and exclusive expression of intron-containing rhodopsin in the pineal gland and the short lifetime of its meta II intermediate, which leads to optimization for pineal photoreception, were achieved after branching of the gar lineage. Based on these results, we propose a stepwise evolutionary model of teleost intron-containing and intron-less rhodopsin genes.

## Introduction

Opsins are photoreceptive molecules that universally underlie the molecular basis of visual and non-visual photoreception in animals^1–3^. Opsins have common structural elements, including seven α-helical transmembrane domains and a chromophore, retinal. Vertebrate rhodopsin is the best-studied opsin that functions as a visual photoreceptor in the retina. Rhodopsin binds 11-*cis*-retinal in the dark, and the photoisomerization of the retinal to the all-*trans* form induces the formation of the meta II intermediate, the active state of rhodopsin, to bind to a G protein, transducin (Gt). Thus, opsins are defined as G protein-coupled receptors (GPCRs) specialized for photoreception. Recent identification and characterization of opsin genes revealed that opsins can be classified into several groups based on their amino acid sequences. This classification corresponds well to the differences of the molecular properties of opsins, such as the photoreaction property and the coupling G protein subtypes. In addition, the phylogenetic classification is strengthened in part by the differences of the exon/intron structures (the number of exons and the boundary positions of exons and introns) of opsin genes^4^. Among the nine human opsin genes, the genes encoding rhodopsin, cone visual pigments and Opn3 are phylogenetically closely related to each other and share the basic exon/intron structures (Fig. S1A). Rhodopsin and blue cone pigment genes have five exons separated by four introns in their coding regions. Red and green cone pigment genes contain an additional intron, and the Opn3 gene lacks one intron. By contrast, the Opn4 and Opn5 genes belong to different opsin groups than vertebrate rhodopsin and have their respective characteristic exon/intron structures: ten exons for the Opn4 gene and seven exons for the Opn5 gene^5,6^. The presence of introns in the human opsin genes is in contrast with the fact that the amino acid sequences of many vertebrate rhodopsin-like GPCRs are encoded by intron-less single-exon genes^7,8^.

Among the opsin genes that have been characterized in various animals, several opsin genes are single-exon genes. For example, it is well known that the coding regions of teleost rhodopsin genes *Rh1* and *Rh1-2* contain no introns^9,10^ (Fig. S1B). The conservation of the exon/intron structures of rhodopsin genes isolated from cyclostomes, cartilaginous fishes and tetrapods suggests the possibility that the teleost intron-less rhodopsin genes emerged by retroduplication near the base of the Actinopterygii^11,12^. A previous analysis of rhodopsin genes in non-teleost fishes revealed partial sequences of these rhodopsin genes and provided an evolutionary model for the appearance of teleost intron-less rhodopsin genes^13^. After that analysis, paralogs of rhodopsin genes were identified in teleosts and were named exo-rhodopsin^14^. The exo-rhodopsin gene (*Exorho*) also shares the five exon/four intron structure and functions exclusively in the teleost pineal gland. Thus, the analysis of both intron-containing and intron-less rhodopsin genes of non-teleost fishes in the Actinopterygii is crucial to reveal the evolutionary origin of the teleost intron-containing and intron-less rhodopsin genes. In this study, we characterized the full-length coding sequences of intron-containing and intron-less rhodopsin genes from major lineages of non-teleost fishes in the Actinopterygii and analyzed their phylogenetic relationship. In addition, we determined their expression patterns in the retina and pineal gland, and the molecular properties of the proteins they encode. Based on these results, we propose the evolutionary history of rhodopsin in the Actinopterygii, including when retroduplication of the rhodopsin gene occurred and when different expression patterns of teleost intron-containing and intron-less rhodopsins emerged.

## Results and Discussion

### Isolation of full-length cDNAs of bichir and sturgeon rhodopsin

**Figure 1:**
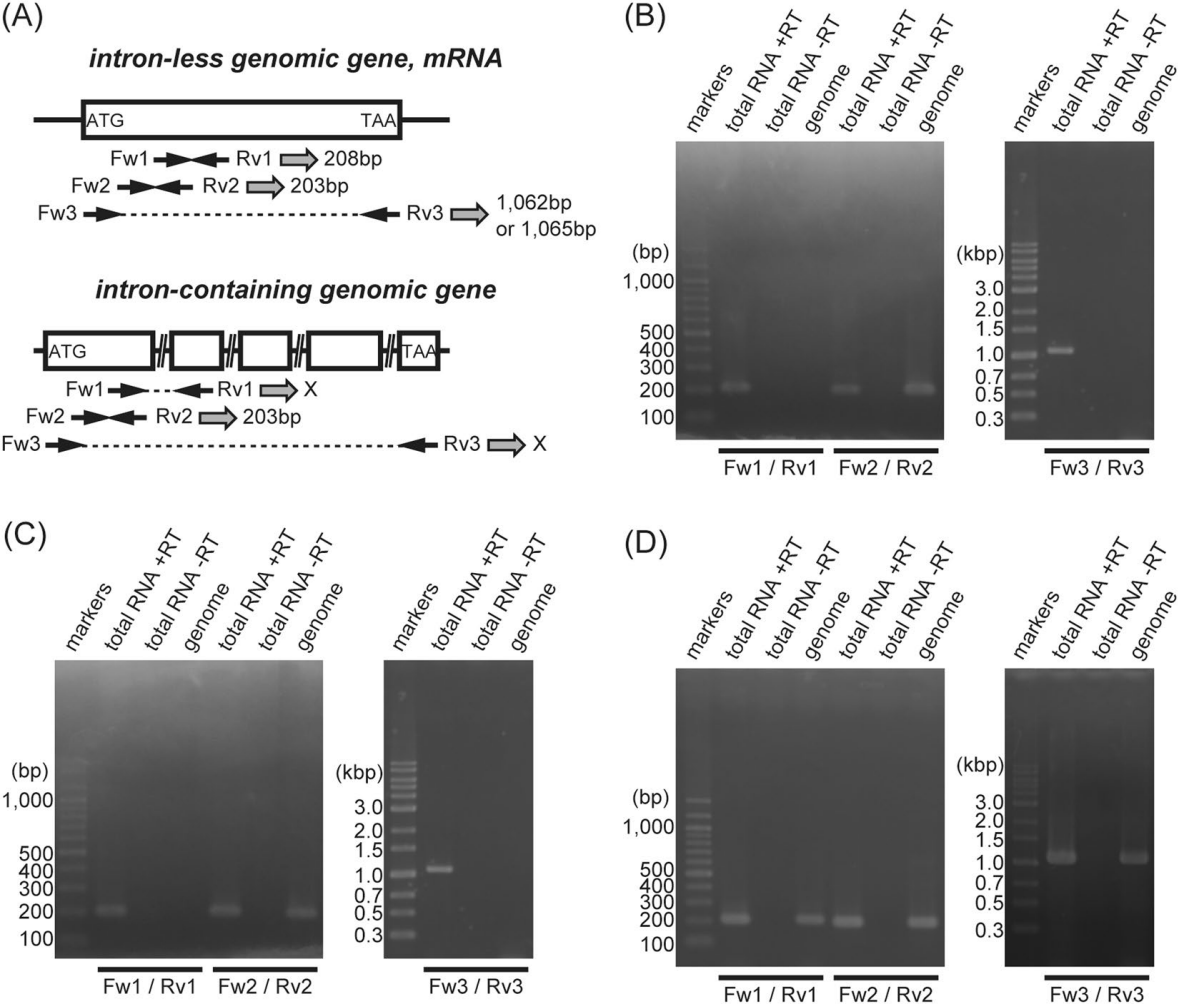
Genomic PCR and RT-PCR analyses of rhodopsin genes. (**A**) Positions of primers for genomic PCR and RT-PCR. We designed three pairs of primers (primers Fw1/Rv1, Fw2/Rv2 and Fw3/Rv3) based on rhodopsin mRNA sequences. Both primers Fw1 and Rv1 target the putative exons 1 and 2, whereas primer Fw2 and Rv2 target the putative exon1, respectively. Thus, if the rhodopsin gene has no introns, a band of around 200 bp can be amplified by the genomic PCR using both primer pairs. If the rhodopsin gene has introns, a band of around 200 bp can be amplified by the genomic PCR using primer pair Fw2/Rv2 but not using primer pair Fw1/Rv1. In addition, primers Fw3 and Rv3 target 5′- and 3′-end of a full-length coding region of the rhodopsin clone, respectively. Thus, if the rhodopsin gene has no introns, a band of around 1,060 bp can be amplified by the genomic PCR using this primer pair. If the rhodopsin gene has introns, a band of around 1,060 bp cannot be amplified by the genomic PCR using this primer pair. Sequences of PCR primers are shown in Table S1. (**B**,**C**) Genomic PCR and RT-PCR on gray bichir (**B**) and reedfish (**C**) rhodopsin. The genomic PCR amplified a band of around 200 bp using only primer pair Fw2/Rv2. It should be noted that RT-PCR on eye total RNA amplified a band of around 200 bp using primer pairs Fw1/Rv1 and Fw2/Rv2 and a band of around 1,060 bp using primer pair Fw3/Rv3. No corresponding bands in the “no reverse transcriptase” reaction confirmed that the band of RT-PCR is not attributed to contaminating genomic DNA. D. Genomic PCR and RT-PCR on Siberian sturgeon rhodopsin. The genomic PCR amplified a band of around 200 bp using primer pairs Fw1/Rv1 and Fw2/Rv2 and a band of around 1,060 bp using primer pair Fw3/Rv3. These bands were also amplified by RT-PCR on eye total RNA, not by “no reverse transcriptase” reaction.

Among non-teleost fishes in the Actinopterygii, spotted gar is the only species whose genomic data are available in the public database. The spotted gar genome contains two rhodopsin genes, an intron-containing gene (*Rh1-1* (*Exorho*)) and an intron-less gene (*Rh1-2*) (Fig. S1B)^15^. First, we searched for full-length rhodopsin cDNAs from gray bichir (*Polypterus senegalus*) and reedfish (*Erpetoichthys calabaricus*) in the Polypteriformes and Siberian sturgeon (*Acipenser baerii*) in the Acipenseriformes. We successfully obtained one full-length rhodopsin cDNA from the eyes of each species and showed the amino acid sequences they encoded in Fig. S2. Next, we examined whether or not these rhodopsin genes have introns (Fig. 1). To perform genomic PCR of gray bichir, reedfish and Siberian sturgeon rhodopsin, we designed three pairs of primers based on their mRNA sequences. First pair (Fw1/Rv1) targets the putative exons 1 and 2, and second pair (Fw2/Rv2) targets the putative exon 1 (Fig. 1A). Thus, if the rhodopsin gene has no introns, a band of around 200 bp can be amplified by the genomic PCR using primer pairs Fw1/Rv1 and Fw2/Rv2. If the rhodopsin gene has introns whose positions are the same as those of tetrapod *Rh1* and teleost *Exorh*, a band of around 200 bp can be amplified by the genomic PCR using primer pair Fw2/Rv2 but not using primer pair Fw1/Rv1. In addition, third pair (Fw3/Rv3) was designed to amplify the full-length ORF of the rhodopsin gene by RT-PCR on eye total RNA (Fig. 1A). The genomic PCR on gray bichir and reedfish rhodopsin showed amplification of a DNA fragment around 200 bp only when using primer pair Fw2/Rv2 (Fig. 1B,C), which indicated that the gray bichir and reedfish rhodopsin genes contain introns. In contrast, the genomic PCR on Siberian sturgeon rhodopsin amplified a band of around 200 bp using primer pairs Fw1/Rv1 and Fw2/Rv2 and a band of around 1,060 bp corresponding to the full-length ORF using primer pair Fw3/Rv3 (Fig. 1D). This showed that the Siberian sturgeon rhodopsin gene has no introns.

**Figure 2:**
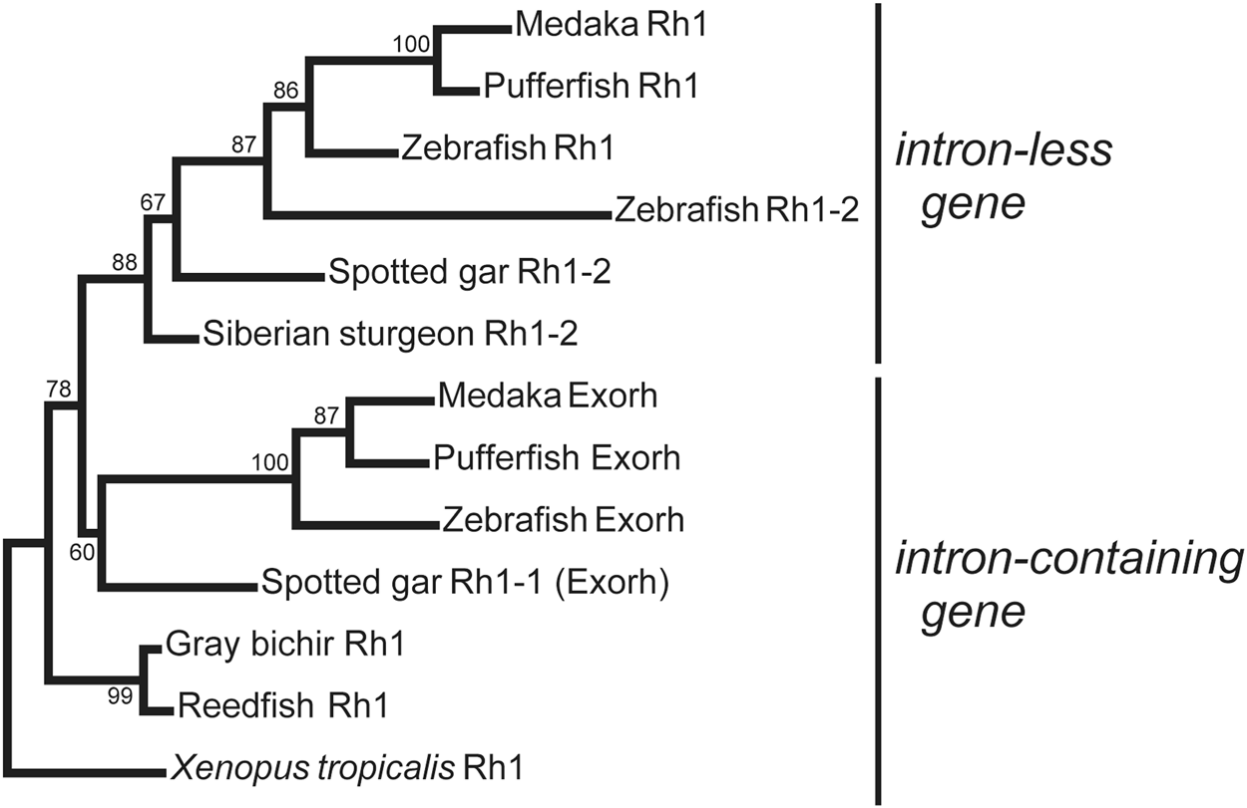
Molecular phylogenetic tree of rhodopsins in the Actinopterygii. The phylogenetic tree was inferred by the maximum-likelihood method (see Methods). The numbers at each node are bootstrap probabilities estimated by 1000 replications. This most likely phylogenetic tree is supported by the synteny analysis of rhodopsin genes of spotted gar and teleosts^12^, which can explain the diversification of *Rh1*, *Rh1-1* (*Exorh*) and *Rh1-2* simply by two gene duplication events. However, the tree contains several branches with not very high bootstrap values. This is probably because there are only a few rhodopsin genes whose full-length sequences are available in non-teleost fishes. The reliability of the tree will be improved by further accumulation of the rhodopsin sequences from non-teleost fishes and will be supported by further synteny analysis of rhodopsin genes in non-teleost fishes. Accession numbers of the sequence data in the tree are as follows: *Xenopus tropicalis* Rh1, BC135234; gray bichir (*Polypterus senegalus*) Rh1, LC438460; reedfish (*Erpetoichthys calabaricus*) Rh1, LC438461; Siberian sturgeon (*Acipenser baerii*) Rh1-2, LC438462; spotted gar (*Lepisosteus oculatus*) Rh1-2, XM_006630625; spotted gar Rh1-1, XM_006630940; zebrafish (*Danio rerio*) Rh1, AF109368; zebrafish Rh1-2, HQ286326; zebrafish Exorh, AB025312; medaka (*Oryzias latipes*) Rh1, AB180742; medaka Exorh, ENSORLG00000010979; pufferfish (*Takifugu rubripes*) Rh1, AF201471; pufferfish Exorh, NM_001033849.

Next, we performed phylogenetic analysis of rhodopsin genes isolated from various species in the Actinopterygii (Fig. 2). The most likely phylogenetic tree revealed that the rhodopsin genes (*Rh1*) from the Polypteriformes (gray bichir and reedfish) are branched from the common ancestor of teleost rhodopsin and exo-rhodopsin, whereas the rhodopsin gene (*Rh1-2*) of Siberian sturgeon in the Acipenseriformes is clustered with teleost rhodopsin genes. Taking account of the evolution of the Actinopterygii (Fig. S1B), these findings suggest that a common ancestral rhodopsin gene (intron-containing gene) in the Actinopterygii was first branched into the intron-containing gene (*Rh1*) in the Polypteriformes and the ancestor of intron-containing (*Rh1-1* (*Exorh*)) and intron-less (*Rh1* and *Rh1-2*) genes by speciation and was subsequently diversified into intron-containing and intron-less genes by retroduplication. Therefore, we speculate that the intron-less rhodopsin genes emerged after the divergence of the Polypteriformes and other Actinopterygii.

In this diversification model, the intron-containing rhodopsin gene (*Rh1-1*) should be present in the sturgeon lineage. However, we could not detect the intron-containing rhodopsin gene in Siberian sturgeon despite detecting intron-less rhodopsin (*Rh1-2*) and green-sensitive cone pigment, which is most closely related to rhodopsin in the phylogenetic tree (Fig. S1A). In addition, we could not find sequence reads corresponding to the intron-containing rhodopsin gene (*Rh1-1*) in the transcriptome data deposited in the NCBI Sequence Read Archive. Thus, we speculate that the intron-containing rhodopsin gene (*Rh1-1*) has been lost in the sturgeon lineage (Fig. S1B).

### Expression patterns of intron-containing and intron-less rhodopsin genes

**Figure 3:**
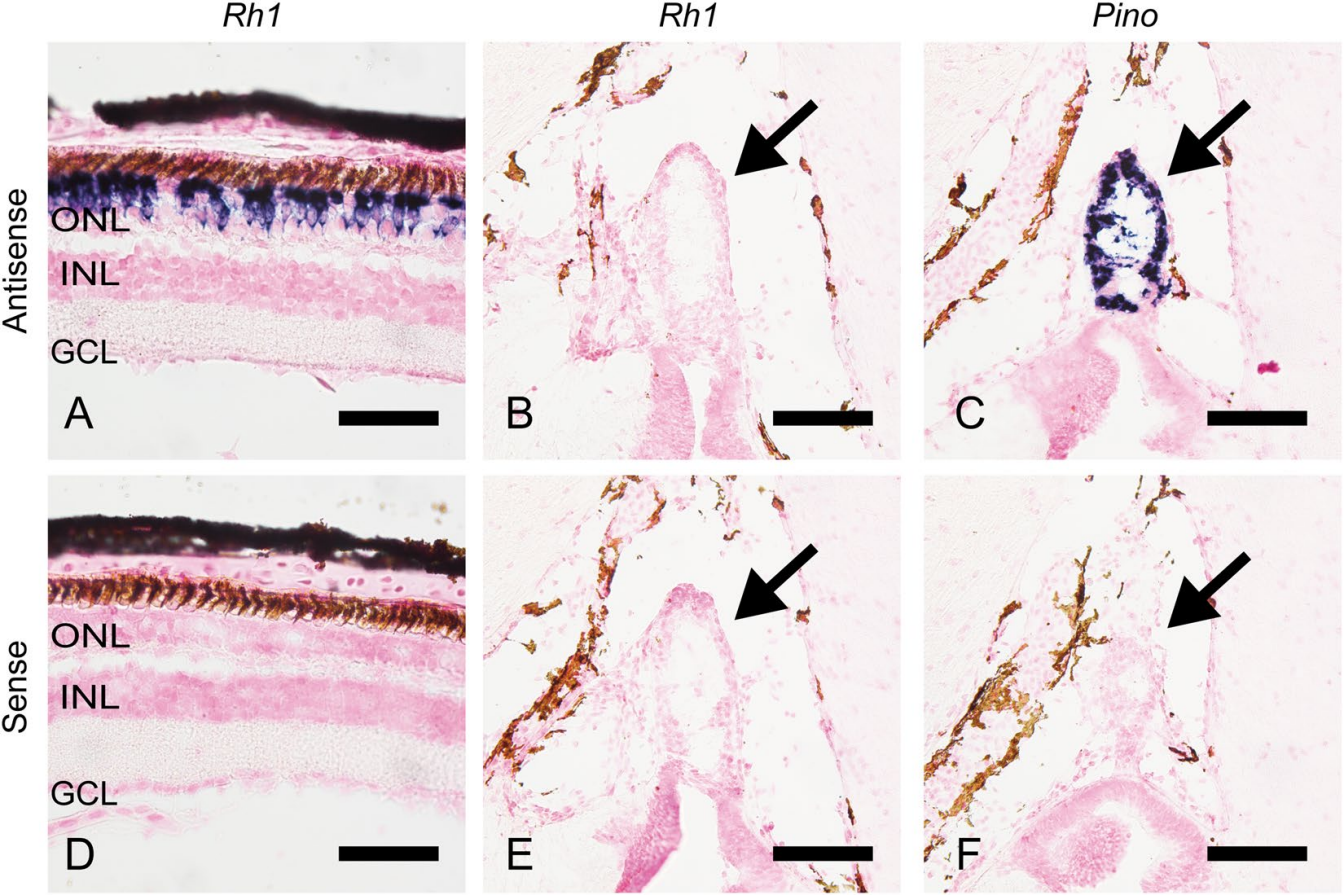
Distribution of rhodopsin in the retina and the pineal gland of gray bichir. *In situ* hybridization of rhodopsin (*Rh1*) and pinopsin (*Pino*) in the retina and the pineal gland of gray bichir. The section of the retina was hybridized with antisense probe of *Rh1* (**A**). The frontal sections of the pineal gland were hybridized with antisense probes of *Rh1* (**B**) and *Pino* (**C**), respectively. (**D–F**) show the consecutive tissue sections to (**A**–**C**) hybridized with corresponding sense probes, respectively. Allows indicate the position of the pineal gland. All the sections shown in this figure were counterstained with Nuclear Fast Red. Scale bar: (**A**,**D**) 50 μm; (**B**,**C**,**E**,**F**) 100 μm. ONL, outer nuclear layer; INL, inner nuclear layer; GCL, ganglion cell layer.

**Figure 4:**
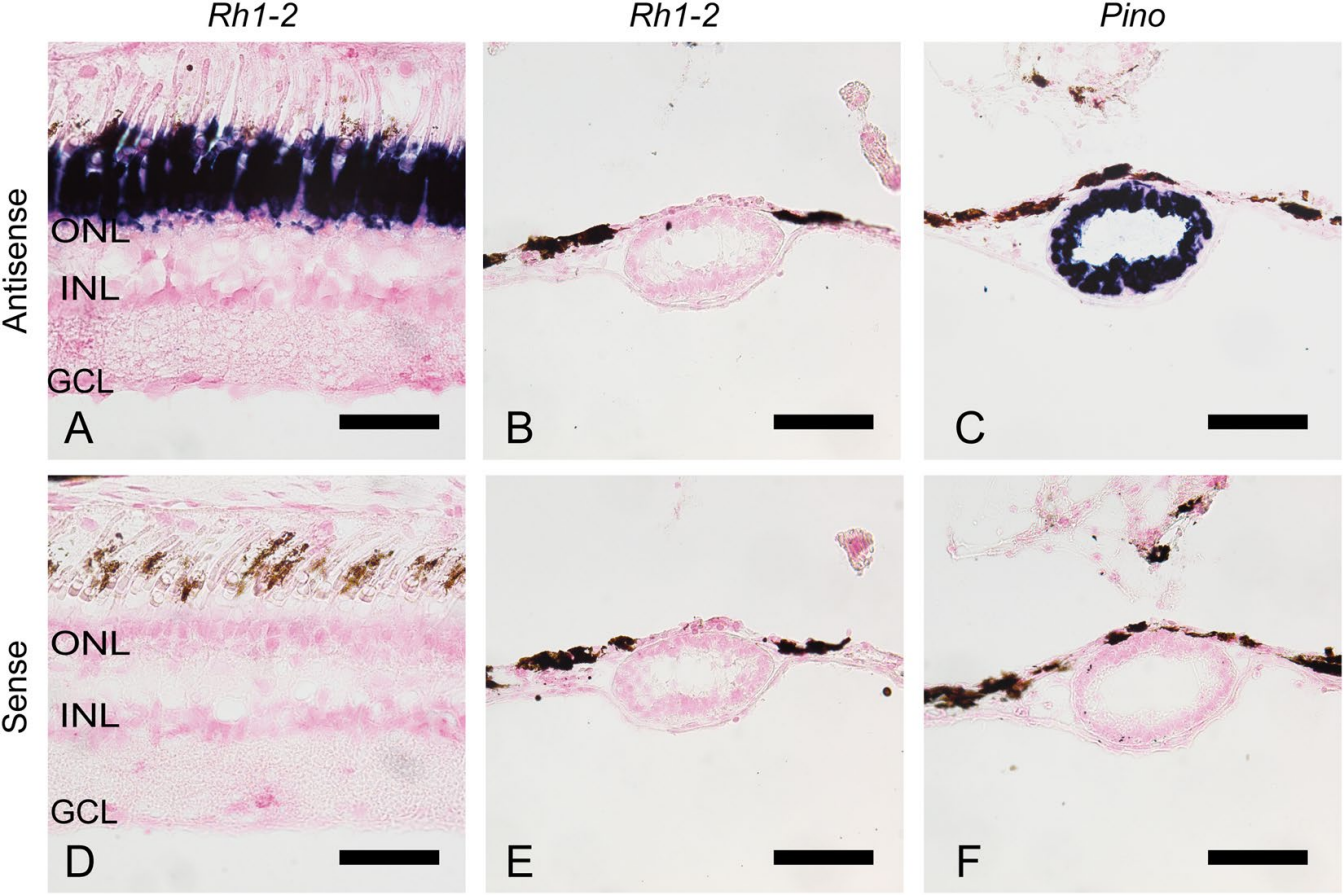
Distribution of rhodopsin in the retina and the pineal gland of Siberian sturgeon. *In situ* hybridization of rhodopsin (*Rh1-2*) and pinopsin (*Pino*) in the retina and the pineal gland of Siberian sturgeon. The section of the retina was hybridized with antisense probe of *Rh1-2* (**A**). The frontal sections of the pineal gland were hybridized with antisense probes of *Rh1-2* (**B**) and *Pino* (**C**), respectively. (**D–F**) show the consecutive tissue sections to (**A**–**C**) hybridized with corresponding sense probes, respectively. All the sections shown in this figure were counterstained with Nuclear Fast Red. Scale bar: (**A**,**D**) 50 μm; (**B**,**C**,**E**,**F**) 100 μm. ONL, outer nuclear layer; INL, inner nuclear layer; GCL, ganglion cell layer.

**Figure 5:**
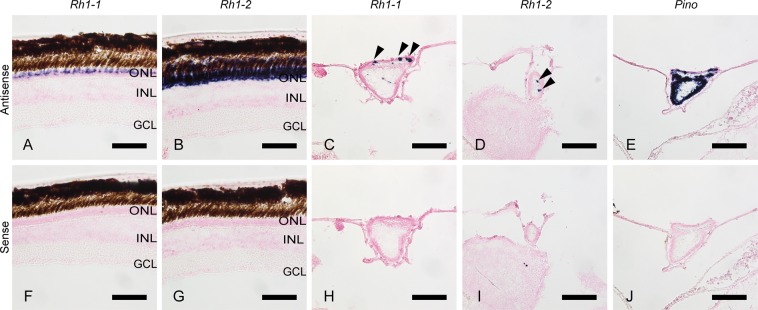
Distribution of rhodopsin in the retina and the pineal gland of spotted gar. *In situ* hybridization of two rhodopsins (*Rh1-1* and *Rh1-2*) and pinopsin (*Pino*) in the retina and the pineal gland of spotted gar. The sections of the retina were hybridized with antisense probes of *Rh1-1* (**A**) and *Rh1-2* (**B**), respectively. The frontal sections of the pineal gland were hybridized with antisense probes of *Rh1-1* (**C**), *Rh1-2* (**D**), and *Pino* (**E**), respectively. Allow heads indicate the positions of hybridization signals of *Rh1-1*in (**C**) and *Rh1-2* in (**D**), respectively. (**F–J**) show the consecutive tissue sections to (**A**–**E**) hybridized with corresponding sense probes, respectively. All the sections shown in this figure were counterstained with Nuclear Fast Red. Scale bar: (**A**,**B**,**F**,**G**) 50 μm; (**C**–**E**,**H**–**J**) 100 μm. ONL, outer nuclear layer; INL, inner nuclear layer; GCL, ganglion cell layer.

It has been reported that the teleost intron-less rhodopsin genes (*Rh1* and *Rh1-2*) and intron-containing rhodopsin gene (*Exorho*) are exclusively expressed in the retina and the pineal gland, respectively^14,16^. We therefore analyzed whether or not gray bichir and Siberian sturgeon rhodopsin genes are expressed in the retina and/or the pineal gland. Our *in situ* hybridization analysis showed that the transcripts of gray bichir intron-containing rhodopsin (*Rh1*) and Siberian sturgeon intron-less rhodopsin (*Rh1-2*) were abundantly expressed in the outer nuclear layer of the retina (Figs 3A and 4A). Moreover, transcripts of these rhodopsins were not observed in the pineal gland of gray bichir or Siberian sturgeon by *in situ* hybridization (Figs 3B and 4B). This contrasts with the strong expression signals of pinopsin in the pineal gland (Figs 3C and 4C), which is consistent with the pinopsin distribution in the pineal-related organs of birds, reptiles and amphibians^17–21^. These expression patterns show that gray bichir and Siberian sturgeon use intron-containing and intron-less rhodopsin genes in their retinas, respectively. Next, we analyzed the expression patterns of intron-containing (*Rh1-1*) and intron-less (*Rh1-2*) rhodopsin genes in the retina and the pineal gland of spotted gar (Fig. 5). Our *in situ* hybridization analysis showed that both rhodopsin genes were expressed in the outer nuclear layer of the retina, as shown in a previous study^22^ and the *Rh1-2*-positive signals were clearly stronger than the *Rh1-1*-positive ones (Fig. 5A,B). Thus, spotted gar predominantly uses an intron-less rhodopsin gene (*Rh1-2*) in the retina. In addition, we observed a minor population of *Rh1-1*-positive cells and a few *Rh1-2*-positive cells in the pineal gland of spotted gar (Fig. 5C,D). However, *Rh1-1*-positive signals were much weaker and sparser than pinopsin-positive signals (Fig. 5E). These weak signals of *Rh1-1* in the spotted gar pineal gland contrast strikingly with the abundant expression of *Exorho* in the teleost pineal gland. These results suggest that the contribution of the intron-containing rhodopsin (*Rh1-1*) gene to pineal photoreception is much less substantial than that of pinopsin in the spotted gar. Therefore, it can be speculated that the abundant and exclusive expression of intron-containing rhodopsin (*Rh1-1*) in the pineal gland was achieved after branching of the gar lineage.

### Molecular property of rhodopsin and exo-rhodopsin

Finally, we analyzed the molecular properties of rhodopsins of non-teleost fishes in the Actinopterygii. We obtained the absorption spectra of the recombinant photopigments of gray bichir rhodopsin (Rh1), reedfish rhodopsin (Rh1), Siberian sturgeon rhodopsin (Rh1-2) and spotted gar rhodopsins (Rh1-1 and Rh1-2) after reconstitution with the 11-cis form of A1 retinal (Fig. S3). All the pigments had the absorption maximum (λmax) at around 500 nm. Gray bichir and reedfish rhodopsins have a unique amino acid residue at position 83, Gly83, which is presumed to have been mutated from the Asp83 or Asn83 conserved among vertebrate rhodopsins. It has been reported that D83G mutation of bovine rhodopsin leads to a small red-shift of λmax^23^. However, contrary to our expectation, gray bichir and reedfish rhodopsins (Rh1) exhibited no detectable spectral shift from 500 nm.

**Figure 6:**
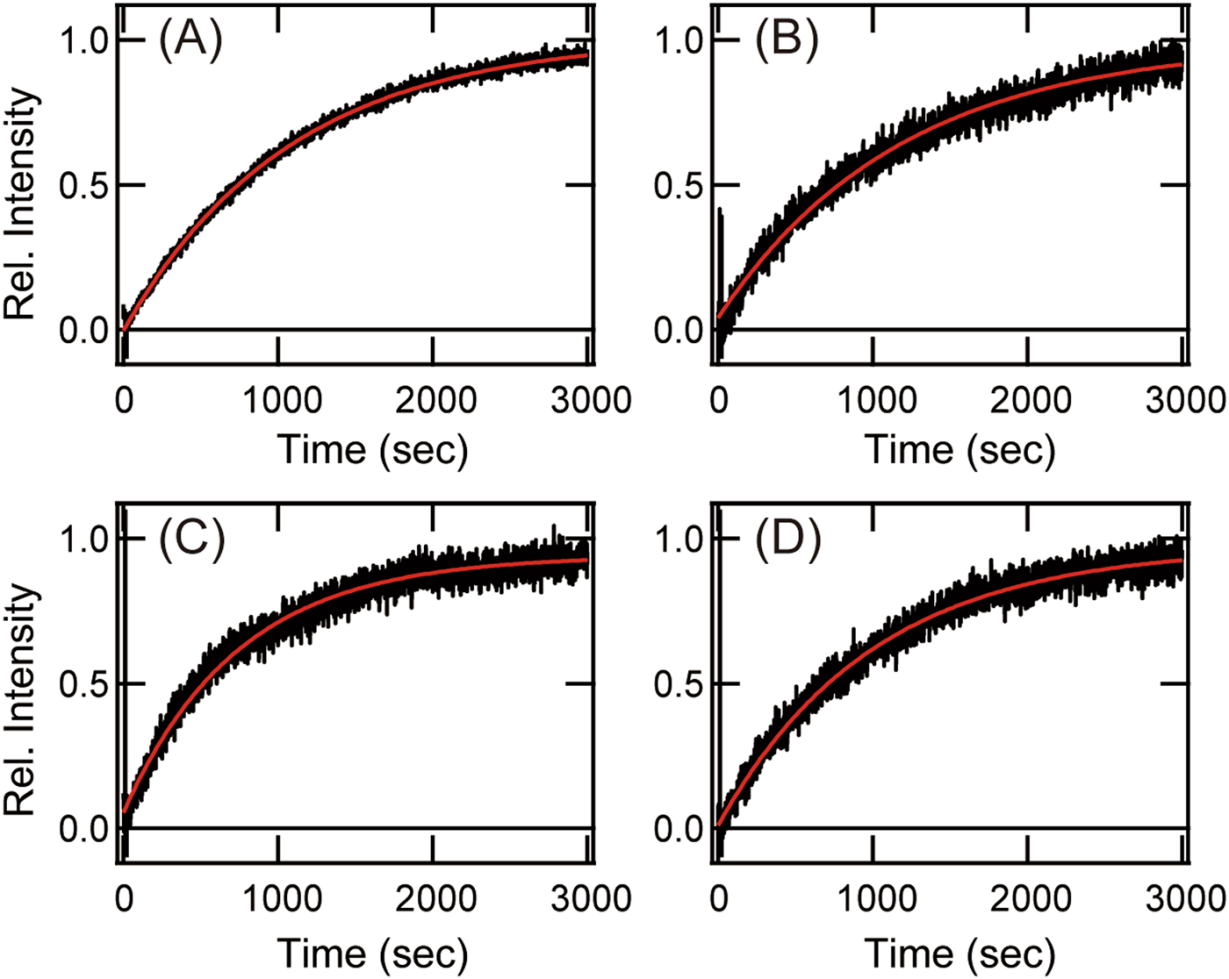
Comparison of the decay of meta II. Decay of meta II of bovine Rh1 (**A**), gray bichir Rh1 (**B**), spotted gar Rh1-2 (**C**) and spotted gar Rh1-1 (Exorh) (**D**) was measured by monitoring the change of intrinsic tryptophan fluorescence after yellow light (>500 nm) irradiation (black line). The data were fitted by a single exponential function (red curve) to estimate the decay rate as follows; bovine Rh1, 875 ± 108 sec; gray bichir Rh1, 913 ± 113 sec; spotted gar Rh1-2, 668 ± 74 sec; spotted gar Rh1-1 (Exorh) 894 ± 129 sec. The average and S.E.M. were calculated based on three independent measurements.

The comparison of the molecular properties of teleost rhodopsin and exo-rhodopsin revealed that the meta II intermediate of exo-rhodopsin decays faster than that of rhodopsin. This is similar to the properties of cone visual pigments, and may contribute to facilitating bleach recovery in the pineal photoreception under continuous bright light conditions^24,25^. Moreover, we recently reported that there is a strong relationship between decay rates of meta II of the visual pigments and thermal isomerization rates of the retinal chromophore of visual pigments in the dark state^26,27^. As a result of this relationship, rhodopsin, whose meta II decays faster, can exhibit a higher thermal activation rate and impair scotopic vision^28,29^. These findings suggest that this alteration of the molecular property of exo-rhodopsin would be associated with restriction of the expression of this opsin to the pineal gland and with a lack of its expression in the rod photoreceptor cells. However, the spotted gar uses both intron-containing and intron-less rhodpsins (*Rh1-1* (*Exorh*) and *Rh1-2*) in the rod photoreceptor cells. Thus, to compare the molecular properties of spotted gar Rh1-1 (Exorh) and Rh1-2 with those of other rhodopsins, we measured the decay rate of meta II of rhodopsins (Fig. 6). We confirmed that meta II of gray bichir Rh1 and spotted gar Rh1-2, which are predominantly distributed in the retina, decays as slowly as that of bovine Rh1. In addition, the decay rate of meta II of spotted gar Rh1-1 (Exorh) is also comparable to that of spotted gar Rh1-2. This shows that spotted gar intron-containing rhodopsin (Rh1-1 (Exorh)) maintains the long lifetime of meta II to function in the rod photoreceptor cells. Therefore, we speculate that the molecular property of intron-containing rhodopsin (Rh1-1 (Exorh)) became optimized for pineal photoreception after branching of the gar lineage.

### Evolutionary model of rhodopsin genes in the Actinopterygii

**Figure 7:**
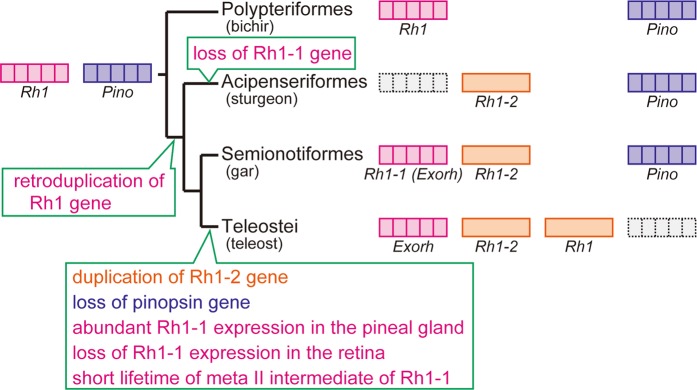
Stepwise evolutionary model of rhodopsin genes in the Actinopterygii. Fishes in the Polypteriformes maintain a single intron-containing rhodopsin gene (*Rh1*) and pinopsin gene like tetrapods. After branching of the Polypteriformes, retroduplication resulted in the formation of intron-less rhodopsin gene (*Rh1-2*). In the lineage of the Acipenseriformes, we speculate that intron-containing *Rh1-1* (*Exorh*) might have been lost probably because of the functional redundancy of rhodopsin and pinopsin in the pineal gland. Fishes in the Semionotiformes maintain two rhodopsin genes (*Rh1-1*(*Exorh*) and *Rh1-2*) and pinopsin gene. By contrast, the ancestor of the Teleostei experienced several events. Teleost-specific whole genome duplication resulted in the formation of *Rh1*, whereas pinopsin gene was lost. Moreover, restricted and abundant expression of *Rh1-1* (*Exorh*) in the pineal gland altered the lifetime of meta II of Rh1-1 (Exorh).

Based on the analysis of rhodopsin genes from non-teleost fishes, we propose an evolutionary model of rhodopsin genes in the Actinopterygii (Fig. 7). The phylogenetic analysis suggested that retroduplication of the rhodopsin gene after branching of the Polypteriformes resulted in the appearance of an intron-less rhodopsin gene (*Rh1-2*). The analysis of the distribution patterns of the expression of rhodopsin genes in the retina and pineal gland revealed that, after branching of the Semionotiformes, the intron-containing rhodopsin gene (*Rh1-1*) changed its expression pattern to exclusive and abundant expression in the pineal gland. The alteration of the distribution pattern of *Rh1-1* (*Exorh*) expression, namely loss of expression in the retina and abundant expression in the pineal gland, would have been associated with the short lifetime of meta II of Rh1-1 (Exorh). Abundant expression of *Rh1-1* (*Exorh*) in the pineal gland may be related to the loss of the pinopsin gene in the teleost lineage. Pinopsin has been found in a wide range of vertebrates except for mammals and teleosts, and is abundantly expressed in the pineal gland of these animals, including non-teleost fishes in the Actinopterygii (Figs 3–5) and cartilaginous fishes (Fig. S4). This suggests the possibility that the common ancestor of the gnathostomes had already acquired the pinopsin gene for pineal photoreception and that the gene was lost from the teleost lineage^19^. Moreover, pinopsin shares common molecular properties with rhodopsin, that is, pinopsin photo-converts to meta II, whose λ_max_ lies in the UV region, to couple with Gt^30^. Because of this functional redundancy of rhodopsin and pinopsin, we speculate that teleosts use the *Rh1-1* (*Exorh*) gene for pineal photoreception instead of the pinopsin gene. By contrast, sturgeons might have maintained the pinopsin gene as a pineal opsin and have lost the *Rh1-1* (*Exorh*) gene. In this study, we did not determine the cis-regulatory elements of rhodopsin genes from the Polypteriformes and the Acipenseriformes. Accumulation of genomic information of these fishes and detailed functional analysis of the cis-regulatory elements of rhodopsin genes will reveal the genetic and evolutionary bases of the stepwise changes of the expression patterns of rhodopsin genes in the Actinopterygii.

## Methods

### Animals and ethics statement

Gray bichir (*Polypterus senegalus*: ~15 cm), reedfish (*Erpetoichthys calabaricus*: ~25 cm), Siberian sturgeon (*Acipenser baerii*: ~20 cm), spotted gar (*Lepisosteus oculatus*: ~7 cm), and coral catshark (*Atelomycterus marmoratus*: ~30 cm) were purchased from a local pet shop. They were euthanized by immobilization using MS222 and immediate decapitation and dissected just after they were brought into our laboratory. The use of animals in these experiments was in accordance with guidelines established by the Ministry of Education, Culture, Sports, Science and Technology of Japan. The protocols in this paper were approved by the Animal Care and Use Committee of Kyoto University (permit number: H2622, H2718 and H2815).

### Isolation of cDNA encoding opsin

The full-length ORF sequences of spotted gar Rh1-1 (Exorh) (XM_006630940), Rh1-2 (XM_006630625) and pinopsin (XM_015367820) and the partial ORF sequence of coral catshark pinopsin (LC328553) were isolated by PCR from the 1st strand cDNA from eyes as shown in our previous study^19^. The full-length cDNA of Siberian sturgeon pinopsin (LC365918) and the partial ORF sequence of gray bichir pinopsin (LC328554) were isolated by PCR from the 1st strand cDNA from brain. To isolate the clone of gray bichir and reedfish rhodopsin, we performed RT-PCR on eye RNA based on the homology to the sequences of lungfish rhodopsin and spotted gar Rh1-1 (Exorh) and Rh1-2. Primer sequences are as follows: 5′-CAACCATGAACGGAACAGAGGG-3′ (forward) and 5′-GCAGGAGAAACCTGGCTGGA-3′ (reverse). We successfully obtained one partial ORF sequence of gray bichir and reedfish rhodopsin. Based on the obtained partial sequence, we performed 5′ and 3′ rapid amplification of cDNA ends by 5′- and 3′-Full RACE Core Set (Takara) to isolate the full-length ORF sequence of gray bichir rhodopsin (LC438460). The full-length cDNA of reedfish rhodopsin (LC438461) was obtained from eyes based on the sequence of gray bichir rhodopsin. Primer sequences are as follows: 5′-TTCTCCTCACGGAAGCCCGG-3′ (forward) and 5′-CCCACGGCGAAGTTGTCTGG-3′ (reverse). To isolate the clone of Siberian sturgeon rhodopsin, we first searched it in the transcriptome data deposited at the NCBI Sequence Read Archive (BioProject accession ID: PRJNA357627). We successfully identified multiple sequence reads corresponding to rhodopsin and isolated the full-length ORF sequence of Siberian sturgeon rhodopsin (LC438462) from eyes. Primer sequences are as follows: 5′-CTCATAAGCAACCGCAACGATG-3′ (forward) and 5′-TTAGCCGCTTTGAAGAGAGG-3′ (reverse). To isolate the clone of Siberian sturgeon intron-containing rhodopsin (Rh1-1(Exorh)), we performed RT-PCR on eye and brain RNA using several pairs of primers, such as 5′-CAACCATGAACGGAACAGAGGG-3′ (forward) and 5′-GCAGGAGAAACCTGGCTGGA-3′ (reverse), which were designed based on the homology to the sequences of spotted gar Rh1-1 (Exorh) and teleost Exorh. Despite of the isolation of Rh1-2 and green-sensitive cone pigment (LC438463), we could not obtain the sequence of Rh1-1 (Exorh). In addition, we searched for the sequence of Siberian sturgeon Rh1-1 (Exorh) in the transcriptome data deposited at the NCBI Sequence Read Archive (BioProject accession ID: PRJNA274436 and PRJNA357627). However, we could not find out sequence reads corresponding to Rh1-1 (Exorh).

### Genomic PCR and RT-PCR analysis

Genomic DNA was isolated from gray bichir, reedfish and Siberian sturgeon using High Pure PCR Template Preparation Kit (Roche). Total RNA was isolated from eyes of gray bichir, reedfish and Siberian sturgeon using RNeasy Plus Mini Kit (QIAGEN). Reverse transcription was performed using oligo dT primer and PrimeScript II reverse transcriptase (Takara). PCR on genomic DNA or 1st strand cDNA was performed for 35 cycles with gene-specific primers (Table S1) using Expand High Fidelity DNA polymerase (SIGMA). Primer target positions were shown in Fig. 1. Each PCR reaction mixture was analyzed on agarose-TAE gel electrophoresis to visualize an amplified band under UV light after staining with ethidium bromide.

### Sequence alignment and molecular phylogenetic analysis

Amino acid sequences of rhodopsins were aligned using ClustalW 2.1^31^. Phylogenetic tree was inferred by maximum-likelihood method. The tree was constructed by MEGA X^32^ using JTT matrix-based model^33^. The sequence alignment of 351 amino acid positions in length excluding gaps was used for the tree inference. The numbers at each node are bootstrap probabilities estimated by 1000 replications.

### Tissue sample preparation

After eyes and brains were dissected from gray bichir, Siberian sturgeon, spotted gar or coral catshark, they were fixed overnight in PBS-buffered 4% PFA at 4 °C. Tissues were subsequently immersed in 20% sucrose in PBS overnight for cryoprotection and were frozen in a deep freezer in OCT compound (tissue tech). Frozen tissues were sliced into 16 μm sections and were attached to glass slides (CREST coat glass slide, Matsunami Glass Co.,Ltd.). Slides were stored in a dry chamber at −20 °C until use.

### *In situ* hybridization

*In situ* hybridization on tissue specimens was carried out as follows. Digoxigenin-labeled RNA probes were synthesized from the cDNAs inserted into pBluescript II KS(+) or pTA2 (TOYOBO). All the following procedures were performed at room temperature unless otherwise noted. Tissue specimens were successively soaked in PBS-buffered 4% PFA for 15 min, 100% methanol for 30 min, PBS for 5 min. After that, the tissue specimens of spotted gar and coral catshark were treated with 0.5 μg/ml proteinase K in Tris buffer (50 mM Tris-HCl, 5 mM EDTA, pH 7.6) for 15 min. For the tissue samples of gray bichir and Siberian sturgeon, this proteinase K treatment step was omitted. Thereafter, specimens were successively immersed in PBS for 5 min, PBS-buffered 4% PFA for 15 min, DEPC-treated water for 2 min, acetylation buffer (0.27% (v/v) acetic anhydride, 0.1 M triethanolamine, pH 8.0) for >10 min, PBS for 5 min, and hybridization buffer (0.75 M NaCl, 75 mM sodium citrate, 0.4 mg/mL yeast RNA (Roche 10109223001), 0.1 mg/mL heparin sodium, 1x Denhardt’s solution, 0.1% (v/v) Tween, 0.1% (w/v) CHAPS, 5 mM EDTA, 70% (v/v) formamide) at 65 °C for 3 hours. After that, digoxigenin-labeled RNA sense or antisense probe diluted with hybridization buffer (final concentration: 0.17 μg/mL) were applied to the specimens and were incubated for about 40 hours at 65 °C. After hybridization, they were successively washed in SSC buffer (0.15 M NaCl, 15 mM sodium citrate, pH 7.0) containing 50% formamide for 15 min and for 1 hour at 65 °C, one-fifth diluted SSC buffer for 1 hour at 65 °C, and MABT (100 mM maleate, NaCl, 0.1% Tween 20, pH 7.5) for three times 30 min. After washing, the specimens were incubated with blocking buffer (1% (w/v) bovine serum albumin, 10% (v/v) sheep normal serum and 0.08% (v/v) Triton-X100 in PBS) for 30 min and were incubated with anti-digoxigenin Fab fragment conjugated with alkaline phosphatase (diluted 1:2000; Roche 11093274910) overnight at 4 °C. The specimens were subsequently rinsed three times with MABT for 30 min, and twice with AP reaction buffer (100 mM Tris-HCl, 50 mM MgCl_2_, 100 mM NaCl, 0.1% Tween 20, pH 9.5) for 5 min. Finally, color development was performed with AP reaction buffer containing 50 μg/ml NBT, 175 μg/ml BCIP and 5% (w/v) polyvinyl alcohol (Sigma-Aldrich 363081).

### Preparation of recombinant proteins

The full-length cDNAs encoding rhodopsin tagged with the epitope sequence of the anti-bovine rhodopsin monoclonal antibody Rho1D4 (ETSQVAPA) at the C terminus were introduced into the mammalian expression vector, pCAGGS^34^ or pMT4^35^. The plasmid DNA was transfected into the HEK293S cell line using the calcium phosphate method. The cell membranes were regenerated with 11-cis form of A1 retinal. The pigments were extracted with Buffer A (50 mM HEPES, 140 mM NaCl, pH 6.5) containing 1% dodecyl maltoside (DDM) and purified using Rho1D4-conjugated agarose. The purified pigments were eluted with 0.02% DDM in Buffer A containing the synthetic C-terminal peptide of bovine rhodopsin.

### UV/Vis absorption spectra and fluorescence measurement

UV/Vis absorption spectra of rhodopsin were recorded using a Shimadzu UV2450 spectrophotometer and an optical cell (width, 2 mm; light path, 1 cm). An optical cell-holder was connected to a Neslab RTE-7 temperature controller, which kept the sample temperature at 0 ± 0.1 °C. Decay of meta II of rhodopsin was measured by monitoring the intrinsic tryptophan fluorescence emission using a Shimadzu RF5300 fluorescence spectrophotometer^36,37^. 60 nM pigments in Buffer A containing 0.02% DDM were irradiated for 10 sec with yellow light (>500 nm) at 20 °C and the change of the fluorescence emission at 340 nm induced by the retinal release was observed. Experimental data were fitted by a single exponential function to estimate the decay rate of meta II.

## Supplementary information

Supplementary information

## Data Availability

Data supporting the findings of this manuscript are available from the corresponding author upon reasonable request.
