# Supplementary material for: Evolutionary history of teleost intron-containing and intron-less rhodopsin genes
Source: Sci Rep. 2019 Jul 23;9:10653. doi: 10.1038/s41598-019-47028-4 (PMC6650399; doi:10.1038/s41598-019-47028-4)
Supplement: Supplementary file 1 — Supplementary information [file 41598_2019_47028_MOESM1_ESM.pdf]

Supplementary Information for

Evolutionary history of teleost intron-containing and intron-less rhodopsin genes

Chihiro Fujiyabu[1], Keita Sato[2], Ni Made Laksmi Utari[2,3], Hideyo Ohuchi[2], Yoshinori Shichida[1,4,5], and Takahiro Yamashita[1,5]

[1]Faculty of Science, Kyoto University, Kyoto 606-8502, Japan; [2]Department of Cytology and Histology, Okayama University Graduate School of Medicine, Dentistry and Pharmaceutical Sciences, Okayama 700-8558, Japan; [3]Department of Ophthalmology, Faculty of Medicine, Udayana University, Bali, Indonesia; [4]Research Organization for Science and Technology, Ritsumeikan University, Shiga 525-8577, Japan; [5]Department of Biophysics, Graduate School of Science, Kyoto University, Kyoto 606-8502, Japan

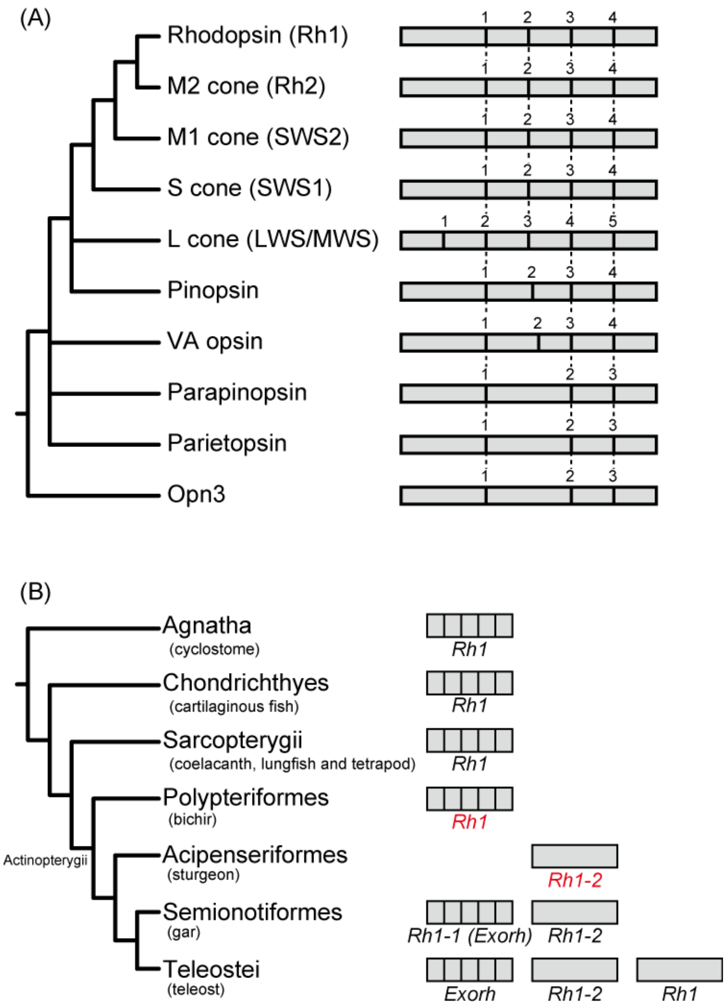

Supplementary Figure 1 Exon/intron structures of vertebrate rhodopsin genes and their related opsin genes

(A) Comparison of the intron positions of opsin genes in the vertebrate visual/non-visual opsin and Opn3 group. The human genome contains four opsin genes, rhodopsin, three cone pigments and Opn3, in this group. Red and green cone pigment genes belong to L cone (LWS/MWS) subgroup and blue cone pigment gene belongs to S cone (SWS1) subgroup. (B) Comparison of the intron-containing and intron-less rhodopsin genes among vertebrates. The nomenclatures of rhodopsin genes refer to those used in previous studies [1-4]. The rhodopsin gene (*Rh1*) of all the vertebrates except for the Actinopterygii has a conserved five exon/four intron structure [5,6]. In zebrafish, the first identified rhodopsin gene, *Rh1*, has no introns in the coding region [3]. Next, an intron-containing rhodopsin gene was identified and was named *Exorh* [7]. Recently this gene was also called *Rh1-1* [1,2,4]. Finally, another intron-less rhodopsin gene (*Rh1-2*) was identified [3]. Many species in the Teleostei have two intron-less rhodopsin genes (*Rh1* and *Rh1-2*) in addition to an intron-containing rhodopsin gene (*Exorh*) [2]. It is speculated that these intron-less rhodopsin genes were acquired by teleost-specific whole genome duplication [2,3]. The genome of spotted gar in the Semionotiformes contains both intron-containing (*Rh1-1 (Exorh)*) and intron-less (*Rh1-2*) rhodopsin genes [8]. In this study, we characterized the full-length rhodopsin cDNAs of gray bichir and reedfish in the Polypteriformes and Siberian sturgeon in the Acipenseriformes (highlighted in red).

```
                                                                               50
Bovine Rh1               MNGTEGPNFY VPFSNKTGVV RSPFEAPQYY LAEPWQFSML AAYMFLLIML GFPINFLTLY VTVQHKKLRT PLNYILLNLA
Gray bichir Rh1          MNGTEGPNFY IPMSNKTGVV RSPFEYPQYY LAEPWKYSAI AAYMFFLIIT AFPINFLTLY VTVQHKKLRS PLNYILLNLA
Reedfish Rh1             MNGTEGPNFY VPMSNKTGVV RSPFDYPQYY LAEPWKYSAI AAYMFFLIIT AFPINFLTLY VTVQHKKLRS PLNYILLNLA
Siberian sturgeon Rh1-2  MNGTEGPDFY VPMSNATGVV RSPFEYPQYY LAEPWKYSAL AAYMFFLILT GFPINFLTLY VTIQHKKLRT PLNYILLNLA
Spotted gar Rh1-2        MNGTEGPNFY VPMSNATGVV RSPFEYPQYY LAEPWKFSAL AAYMFFLIIT GFPINFLTLY VTIEHKKLRT PLNYILLNLA
Zebrafish Rh1            MNGTEGPAFY VPMSNATGVV RSPYEYPQYY LVAPWAYGLL AAYMFFLIIT GFPVNFLTLY VTIEHKKLRT PLNYILLNLA
Zebrafish Rh1-2          MNGTEGPDFY VPMSNESGVV RSPYEYPQYY LASPFAFYCM AAYMLFLIVT CVPVNGLTLY VTIENKKLRT PLNYILLNLA
Spotted gar Rh1-1        MNGTEGPNFY VPMSNKTGIV KNPFEYPQYY LAEPWKFSAL AAYMFFLIIT GFPINFLTLH VTIQHKKLRT PLNYILLNLA
Zebrafish Exorh          MNGTEGPNFY VPMSNRTGLV RSPFEEPQYY LAEPWQFSLL AAYMLFLILG SFPINALTLY VTVQHKKLRT PLNYILLNLA

                                   100                                                    150
Bovine Rh1               VADLFMVFGG FTTTLYTSLH GYFVFGPTGC NLEGFFATLG GEIALWSLVV LAIERYVVVC KPMSNFRFGE NHAIMGVAFT
Gray bichir Rh1          VSGLFMVFGG FTTTLYTSMH GYFIFGETGC NLEGFFATLG GEIGLWSLVV LAIERYIVVC KPMANFRFGE NHAIMGVVFT
Reedfish Rh1             VSGLFMVFGG FTTTLYTSMH GYFVFGETGC NLEGFFATLG GEIGLWSLVV LAVERYIVVC KPMANFRFGE NHAIMGVVFT
Siberian sturgeon Rh1-2  VADLFMVFGG FTTTMYTSMN GYFVFGTTGC NIEGFFATLG GEIGLWCLVV LAIERYIVVC KPMSNFRFGE NHAIMGVVFT
Spotted gar Rh1-2        VGDLFMVFGG FTTTMYTSMN GYFVFGTTGC NIEGFFATLG GMISLWSLVV LAVERYLVVC KPMSNFRFGE SHSIMGVLFT
Zebrafish Rh1            IADLFMVFGG FTTTMYTSLH GYFVFGRLGC NLEGFFATLG GEMGLWSLVV LAIERWMVVC KPVSNFRFGE NHAIMGVAFT
Zebrafish Rh1-2          VADLFMVFGG FTTTFYTSMH GYFVLGRAGC NLEGLFATVG GEIALWSLVV LAVERWVVVC KPFTKFRFSQ LHATLGVAFS
Spotted gar Rh1-1        VADLFMITGG FTTTMYTSMH GYFVFGPAGC NVEGFFATLG GEIALWSLVV LAIERYIVVC KPVSNFRFGE NHAVMGVALT
Zebrafish Exorh          VADLFMVLGG FTVTLYTALH GYFLLGVTGC NIEGFFATLG GEIALWSLVV LAIERYIVVC KPMSTFRFGE KHAIIGVGFT

                                                          200
Bovine Rh1               WVMALACAAP PLVGWSRYIP EGMQCSCGID YYTPHEETNN ESFVIYMFVV HFIIPLIVIF FCYGQLVFTV KEAAAQQQES
Gray bichir Rh1          WIMALSCAAP PLLGWSRYIP EGMQCSCGID YYTLKPEVNN ESFVIYMFIV HFTIPMIVIF FCYGRLVCTV KEAAAQQQES
Reedfish Rh1             WIMALSCAAP PLLGWSRYIP EGMQCSCGID YYTLKPEVNN ESFVIYMFIV HFSIPMVVIF FCYGRLVCTV KEAAAQQQES
Siberian sturgeon Rh1-2  WIMALSCAAP PLFGWSRYIP EGMQCSCGID YYTLKPEVNN ESFVIYMFVV HFFIPLIIIS FCYGRLVCTV KEAAAQQQES
Spotted gar Rh1-2        WVMACACAVP PLFGWSRYIP EGMQCSCGID YYTLKPEVNN ESFVIYMFTV HLLLPFSVVF FCYGRLVCTV KAAAAQQQES
Zebrafish Rh1            WVMACSCAVP PLVGWSRYIP EGMQCSCGVD YYTRTPGVNN ESFVIYMFIV HFFIPLIVIF FCYGRLVCTV KEAAAQQQES
Zebrafish Rh1-2          WSMACSCAIP PLLGWSRYIP EGLQCSCGVD YYTPNPETEN ESFVIYMFVV HFSIPLTIIS FCYGRLLCTV KVAAAQQQES
Spotted gar Rh1-1        WIMALACAAP PLFGWSRYIP EGMQCSCGID YYTVTPEVNN KSFVIYMFIV HFSIPLTVIF FCYGRLVCTV KQAASQQQES
Zebrafish Exorh          WVMALTCAVP PLLGWSRYIP EGMQCSCGID YYTPKPEVHN TSFVIYMFIL HFSIPLLIIF FCYSRLLCTV RAAAAQQQES

                                   250                                                    300
Bovine Rh1               ATTQKAEKEV TRMVIIMVIA FLICWLPYAG VAFYIFTHQG SDFGPIFMTI PAFFAKTSAV YNPVIYIMMN KQFRNCMVTT
Gray bichir Rh1          ETTQRAEREV TRMVVVMVVG FLICWVPYAS VAWYIFTHQG TDFGPVFMTA PAFFAKSASL YNPIIYIFLN KQFRHCMITT
Reedfish Rh1             ETTQRAEREV TRMVVVMVVG FLICWLPYAS VAWYIFTHQG ADFGPVFMTA PAFFAKSASL YNPIIYIFLN KQFRHCMITT
Siberian sturgeon Rh1-2  ETTQRAEREV TRMVVLMVIS FLVCWTPYAS VAWYIFTHQG SHFGPVFMTT PAFFAKSSAL YNPLIYILMN KQFRHCMITT
Spotted gar Rh1-2        ETTQRAEREV TRMVVLMVIS YIVCWTPYGS VAWYIFTHKG ADFGPVFMTG PAFFAKSSAL YNPLIYVCMN KQFRHCMITT
Zebrafish Rh1            ETTQRAEREV TRMVIIMVIA FLICWLPYAG VAWYIFTHQG SEFGPVFMTL PAFFAKTSAV YNPCIYICMN KQFRHCMITT
Zebrafish Rh1-2          ETTQRAEREV TRMVILMVIA FLICWLPYAS VAWYIFTHQG SQFGPVFMTV PAFFAKSSAL YNPLIYVFMN KQFRHSMMMT
Spotted gar Rh1-1        ETTQRAEKEV TRMVVVMVIS FLVCWLPYAS VAFYIFVNQG SNFGPVFMTA PAFFAKSSAL YNPIIYILLN KQFRRCMITT
Zebrafish Exorh          ETTQRAEREV TRMVVVMVIA FLVCWVPYAS VAWYIFANQG AEFGPVFMTV PAFFAKSAAL YNPVIYIMLN RQFRNCMLST

                                              348
Bovine Rh1               LCCGKNPLG- DDEASTTVS- KTETS----- QVAPA
Gray bichir Rh1          LCCGKNPFG- EEDGTSAASS KTEASSVSSS QVSPA
Reedfish Rh1             LCCGKNPFG- EEDGTSAASS KTEASSVSSS QVSPA
Siberian sturgeon Rh1-2  LCCGKNPFE- EEEG-SVSAS KTEASSVSSS SVSPA
Spotted gar Rh1-2        LCCGKNPFE- EEEGASTTAS KTEASSVSSS QVSPA
Zebrafish Rh1            LCCGKNPFE- EEEGASTTAS KTEASSVSSS SVSPA
Zebrafish Rh1-2          VCCGKDPFQD EEEGSSSSKS KTETSSVSSS SASSA
Spotted gar Rh1-1        LCCGKNPFG- EED-TTSASG KTEASSVSSS QVSPA
Zebrafish Exorh          VCCGKNPLA- EDESSSAVSS KTQSSVVSSA QVSPA
```

Supplementary Figure 2 Comparison of the amino acid sequences of rhodopsins in the Actinopterygii

The numbers indicate amino acid residue positions of bovine Rh1. Accession numbers of the sequence data are as follows: bovine Rh1, AH001149; gray bichir (*Polypterus senegalus*) Rh1, LC438460; reedfish (*Erpetoichthys calabaricus*) Rh1, LC438461; Siberian sturgeon (*Acipenser baerii*) Rh1-2, LC438462; spotted gar (Lepisosteus oculatus) Rh1-2, XM_006630625; spotted gar Rh1-1, XM_006630940; zebrafish (*Danio rerio*) Rh1, AF109368; zebrafish Rh1-2, HQ286326; zebrafish Exorh, AB025312.

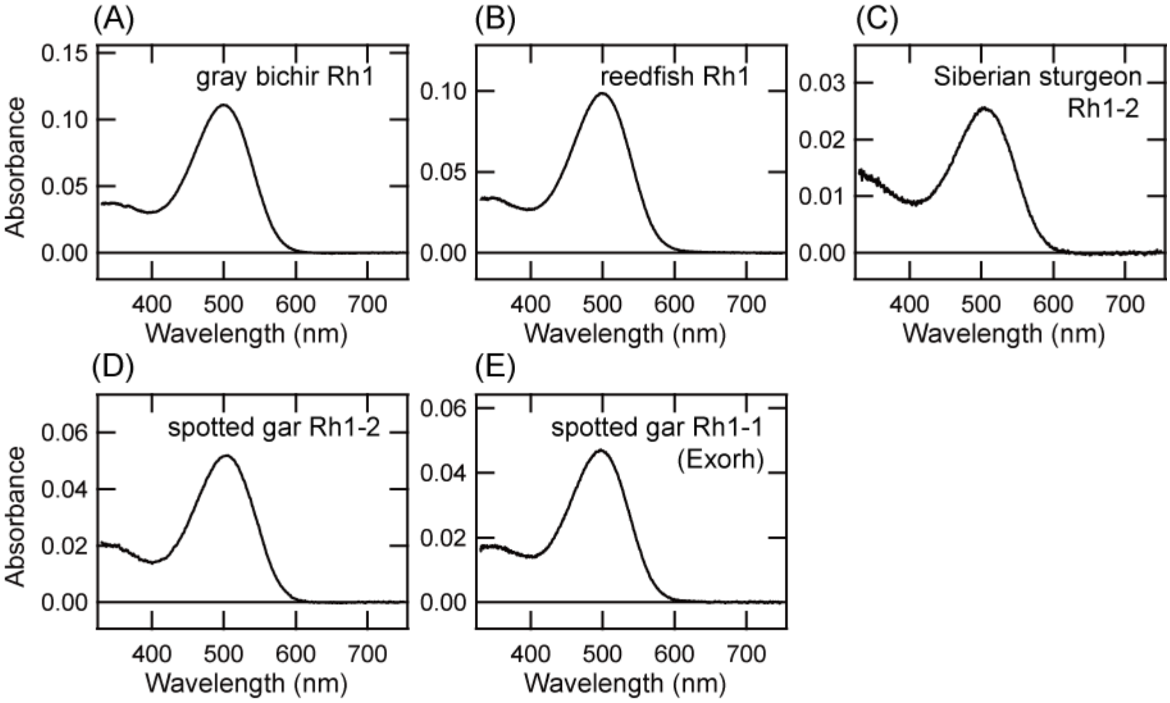

Supplementary Figure 3 Absorption spectra of rhodopsins

Absorption spectra of gray bichir Rh1 (A), reedfish Rh1 (B), Siberian sturgeon Rh1-2 (C), spotted gar Rh1-2 (D) and spotted gar Rh1-1 (Exorh) (E) were measured after reconstitution with 11-*cis*-form of A1 retinal. All the pigments exhibited almost the same λmax at around 500nm. It should be noted that many fishes use both A1 and A2 retinal as a chromophore of the pigments [9]. Thus, rhodopsins of these fish species can exhibit red-shifted λmax by the incorporation of 11-cis form of A2 retinal.

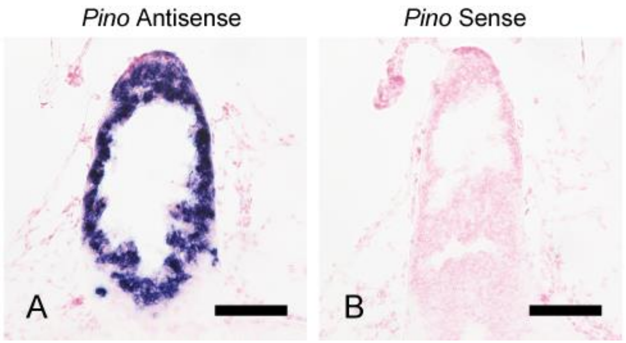

Supplementary Figure 4 Distribution of pinopsin in the pineal gland of coral catshark (*Atelomycterus marmoratus*)

Transcript of pinopsin (*Pino*) was detected by in situ hybridization in the pineal gland of coral catshark. The frontal consecutive sections of the pineal gland were hybridized with antisense (A) and sense (B) probe of *Pino*, respectively. All the sections were counterstained with Nuclear Fast Red. Scale bar: 100 μm.

Supplementary Table 1 List of primers used for genomic PCR and RT-PCR analyses in Fig. 1

species	primer name	sequence
gray bichir and reedfish	Fw1	TGGCAGTGTCCGGCTTATTC
	Rv1	GAAGTTAGCCATAGGTTTGC
	Fw2	GAGCCATGGAAGTACTCTGC
	Rv2	AGCCATGCATGGATGTGTAC
	Fw3	ATGAACGGAACAGAGGGTCC
	Rv3	TTACGCAGGAGACACCTGGC
Siberian sturgeon	Fw1	TGGCTGTGGCTGATCTGTTC
	Rv1	GAAGTTGCTCATGGGCTTGC
	Fw2	GAACCATGGAAGTATTCGGC
	Rv2	AGCCATTCATCGATGTGTAC
	Fw3	ATGAACGGCACAGAGGGTCC
	Rv3	TTATGCTGGAGAGACAGAAC

Supplementary references

1 Beaudry, F. E. G. *et al.* The non-visual opsins: eighteen in the ancestor of vertebrates, astonishing increase in ray-finned fish, and loss in amniotes. *J Exp Zool B Mol Dev Evol* **328**, 685-696, doi:10.1002/jez.b.22773 (2017).

2 Lin, J. J., Wang, F. Y., Li, W. H. & Wang, T. Y. The rises and falls of opsin genes in 59 ray-finned fish genomes and their implications for environmental adaptation. *Sci Rep* **7**, 15568 (2017).

3 Morrow, J. M., Lazic, S. & Chang, B. S. A novel rhodopsin-like gene expressed in zebrafish retina. *Vis Neurosci* **28**, 325-335 (2011).

4 Sukeena, J. M. *et al.* Characterization and Evolution of the Spotted Gar Retina. *J Exp Zool B Mol Dev Evol* **326**, 403-421, doi:10.1002/jez.b.22710 (2016).

5 Bellingham, J. & Foster, R. G. Opsins and mammalian photoentrainment. *Cell Tissue Res* **309**, 57-71, doi:10.1007/s00441-002-0573-4 (2002).

6 Fitzgibbon, J. *et al.* The rhodopsin-encoding gene of bony fish lacks introns. *Gene* **164**, 273-277 (1995).

7 Mano, H., Kojima, D. & Fukada, Y. Exo-rhodopsin: a novel rhodopsin expressed in the zebrafish pineal gland. *Brain Res Mol Brain Res* **73**, 110-118 (1999).

8 Lagman, D. *et al.* The vertebrate ancestral repertoire of visual opsins, transducin alpha subunits and oxytocin/vasopressin receptors was established by duplication of their shared genomic region in the two rounds of early vertebrate genome duplications. *BMC Evol Biol* **13**, 238, doi: 10.1186/1471-2148-13-238 (2013).

9 Dartnall, H. J. & Lythgoe, J. N. The spectral clustering of visual pigments. *Vision Res* **5**, 81-100 (1965).
